# A database for global soil health assessment

**DOI:** 10.1038/s41597-020-0356-3

**Published:** 2020-01-13

**Authors:** Jinshi Jian, Xuan Du, Ryan D. Stewart

**Affiliations:** 1 School of Plant and Environmental Sciences, Virginia Tech, Blacksburg, VA USA; 2 Pacific Northwest National Laboratory-University of Maryland Joint Global Change Research Institute, 5825 University Research Court, Suite 3500, College Park, MD USA; 3 Department of Hydraulic Engineering, Yangling Vocational & Technical College, Yang Ling, Shaanxi China

**Keywords:** Carbon cycle, Environmental impact, Agriculture

## Abstract

Field studies have been performed for decades to analyze effects of different management practices on agricultural soils and crop yields, but these data have never been combined together in a way that can inform current and future cropland management. Here, we collected, extracted, and integrated a database of soil health measurements conducted in the field from sites across the globe. The database, named *SoilHealthDB*, currently focuses on four main conservation management methods: cover crops, no-tillage, agro-forestry systems, and organic farming. These studies represent 354 geographic sites (i.e., locations with unique latitudes and longitudes) in 42 countries around the world. The *SoilHealthDB* includes 42 soil health indicators and 46 background indicators that describe factors such as climate, elevation, and soil type. A primary goal of this effort is to enable the research community to perform comprehensive analyses, e.g., meta-analyses, of soil health changes related to cropland conservation management. The database also provides a common framework for sharing soil health, and the scientific research community is encouraged to contribute their own measurements.

## Abstract (summary)

**Table:** 

Measurement(s)	organic material • grain yield trait • mass density of soil • concentration of carbon atom in soil • soil organic carbon sequestration rate • concentration of nitrogen atom in soil • phosphorus • potassium • pH measurement • soil cation exchange capability • electrical conductivity • soil base saturation • aggregate stability • porosity of soil • soil penetration resistance • soil infiltration rate • field saturated hydraulic conductivity • soil erosion • flood • soil nutrient leaching • temperature of soil • soil water content • available water holding capacity • parasitic weed • diseases of the cropland • pests • Lumbricus terrestris • Arthropoda • Nematoda • Bacteria • Fungi • mycorrhiza • enzyme activity • dinitrogen oxide • carbon dioxide • methane gas emission process
Technology Type(s)	digital curation
Factor Type(s)	conservation management method
Sample Characteristic - Environment	soil environment
Sample Characteristic - Location	United States of America • Canada • Germany • Brazil • French Republic • Italy • Sweden • Kingdom of Denmark • Nigeria • Argentina • Kenya • Zimbabwe • China • South Korea • Kingdom of Spain • Kingdom of the Netherlands • Greece • Kingdom of Norway • New Zealand • Russia • Australia • Ghana • Malawi • Benin • Zambia • Cameroon • Peru • Indonesia • The Philippines • Rwanda • Uganda • Togo • Guinea • Tanzania • Turkey • Moldova • England • India • Costa Rica • Switzerland • Bangladesh • Poland

Machine-accessible metadata file describing the reported data: 10.6084/m9.figshare.10748210

## Background & Summary

Soil health, sometimes used interchangeably with soil quality, represents the ability of soils to function as a biodiverse organism that sustains terrestrial life (USDA-NRCS, 2019), and is often assessed using a combination of physical, chemical and biological indicators^1^. Cropland soil degradation due to natural vegetation removal, intensive agricultural operations, and erosion are among the main factors causing declines in soil health and crop yields^2–4^. According to a recent report from the Food and Agriculture Organization of the United Nations (FAO), one-third of soils in the world are infertile due to unsustainable land-use management practices^5^. Cropland conservation management practices, including the use of cover crops within rotations and changes from traditional mouldboard or disk tillage to reduced or no-tillage, have been proposed as ways to increase soil carbon and soil health^6,7^. Many on-site experiments have been conducted to evaluate the effects of conservation management on soil properties, yet there has been little effort to evaluate which indicators should be measured to consistently quantify any resulting improvements in soil health. In addition, studies can differ in their results: as an example, using cover crops during normally fallow seasons can enhance soil organic carbon^8^, though many short-term studies have not found this same result^9–11^.

To better address such uncertainties, systematic reviews and meta-analyses have evaluated the effects of cover crops^12^, no-tillage^13,14^, organic farm^15^, and agroforestry systems^16^ on crop yield and soil properties. These efforts have generated new insights into soil health dynamics, yet there is still limited understanding of whether and how these findings translate to global scales. Historically and newly published data offer a wealth of information that can support global assessments of how conservation agricultural practices may influence soil health, provided that there is an effective mechanism to record and disseminate this information.

To address this gap, we collected studies that compared agricultural production and soil properties under traditional management strategies with those under conservational management practices. Publications that meet specific criteria were digitized and the data were integrated into a global soil health database that we have named *SoilHealthDB*. This web-based, open source dataset can be continuously updated by including newly published and even provisional data. The dataset can be used to perform statistical analyses (e.g., meta-analyses) on specific soil health indicators or agronomic responses. *SoilHealthDB* provides a common soil health framework for sharing and integrating field measurements and related information, and thereby offers valuable information for farmers, agency personnel, and scientists as they plan and evaluate cropland management.

## Methods

### Data collection

*SoilHealthDB* currently includes 46 background indicators (Online-only Table 1) and 42 soil health indicators (Online-only Table 2)^1^. To identify relevant studies, we conducted a systematic literature search for field comparisons between traditional and conservational management practices. We initially targeted four main conservational management methods: cover cropping (CC), no-tillage (NT), organic farming (OF), and agro-forestry systems (AF) (Table 1).

**Table 1:** Conservation type included in *SoilHealthDB*.

Conservation type	Description
Cover crop (CC)	In conventional row crop farming systems, the soil surface often is left bare after harvesting and thus may cause soil erosion, leaching, and decreases in SOC^2–4^. A cover crop is a plant grown during the fallow season. Grasses or legumes are the major types of cover crops but other green plants such as brassicas are also used. Cover crops are grown primarily for benefit of the soil rather than for crop yield, though cash crop yield increases can result from this practice^28^.
No-tillage (NT)	No-tillage (also named no-till, zero tillage, and direct drilling) is a way of growing crops with minimal soil disturbance. Benefits of no-tillage include: reduced soil erosion, runoff, and leaching; improved soil infiltration; and increased soil organic carbon^14^.
Agriculture forest system (AF)	Agriculture forest system (also called agro-forestry) is a farmland management practice that combines trees or shrubs with crops or pastures. Benefits of agriculture forest systems include prevention of soil erosion and increased biodiversity. In sub-Saharan Africa and in parts of the United States, agriculture forest systems have been successfully applied^16^.
Organic farming (OF)	Organic farming uses organic fertilizers (e.g., compost manure, green manure, and bone meal) rather than inorganic chemical fertilizers and pesticides. Organic farming can lead to increased soil carbon concentrations^15^.

Publications were searched and collected from three sources: (1) an online literature search; (2) the Soil Health Institute “Research Landscape Tool”, which compiles soil health results into a searchable database and includes publication and research projects^17^; and (3) cited papers from previous meta-analyses or review papers^12,15,18,19^. For the online literature search we used the ISI Web of Science, Google Scholar, and the China National Knowledge Infrastructure (CNKI). We used the keywords “soil health” or “soil quality” and “conservation management”, “cover crop”, “no-till”, “organic farm”, or “agroforestry systems” when performing the literature search. Papers from peer-reviewed journals, conference collections, theses, and dissertations were included. No other restrictions or filtering criteria were used (e.g., we included eligible papers in all languages and with all publication dates). We collected a total of more than 500 papers; we then used the following criteria to determine whether the publication would be included in this study: (1) experiments were conducted in the field or at a research station; (2) the publications compared controls (i.e., traditional management) and treatments (i.e., conservational management); (3) publications provide at least one comparison of soil health indicators between controls and treatments (Online-only Table 2). Within these constraints, 321 papers were extracted and integrated into the *SoilHealthDB*.

**Fig. 1:**
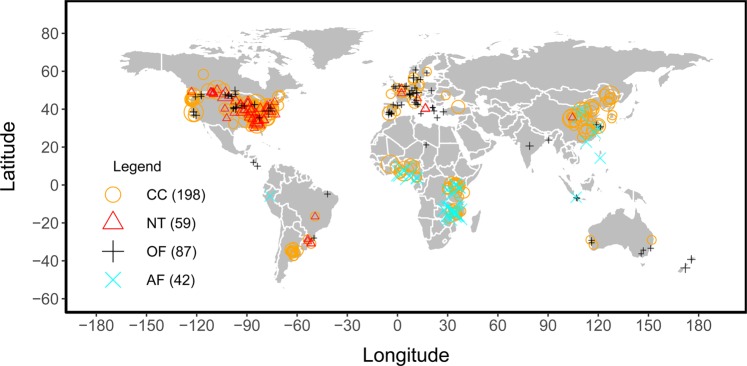
The spatial distribution of sites from cover cropping (CC), no-tillage (NT), organic farming (OF), and agro-forestry systems (AF) across the globe. The numbers in the parentheses represent the number of sites reporting data for each different conservation management method. Symbol sizes represent the number of comparisons in each site.

**Fig. 2:**
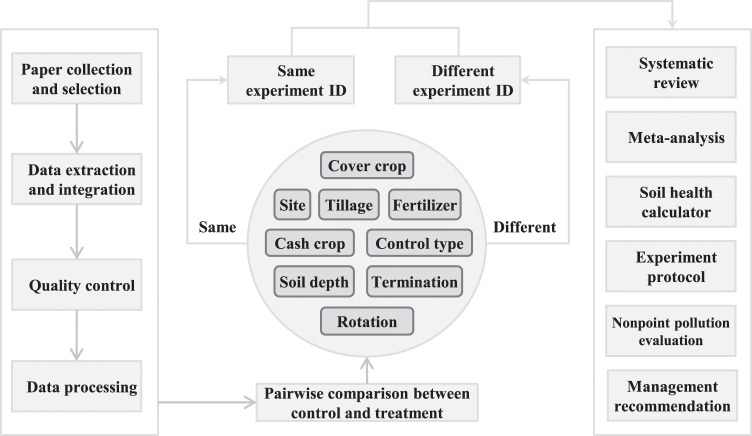
Diagram detailing the procedures for data integration, experiment ID allocation, and potential uses that the database can support. Unique experiment IDs were given to pairwise comparisons if the cash crop, site, tillage, fertilizer level, cover crop, soil sampling depth, cover crop termination, or cash crop rotation was different from other comparisons; otherwise, comparisons that had the same information for one or more of those categories received the same experiment ID (middle panel).

Data were digitized from tables and figures. The software Data Thief (version III)^20^ was used to read the data from figures. Background information was extracted from the publications and fit into 46 background indicator categories (Online-only Table 1). Whenever latitude and longitude were not reported in the literature, the site name was entered into the website (https://www.findlatitudeandlongitude.com) to estimate location. Whenever elevation was missing from the original paper, it was identified by latitude and longitude (https://www.freemaptools.com/elevation-finder.htm). In total, 5,907 comparisons were collected from across the globe (Fig. 1), for a mean of approximately 20 comparisons per study. As many studies reported multiple comparisons, we needed to identify if those comparisons were independent of one another. We therefore allocated a unique experiment ID to a comparison if the cover crop group, cash crop group, site, tillage, fertilization, soil depth, termination, or rotation were different from other comparisons (Fig. 2). This process resulted in a total of 1,407 experiments that were assumed to be independent of each other.

### Data processing

After the location information was carefully checked, the climatic regions for all sites were identified according to climate Koppen classification^21^, using the latitude and longitude (for a detailed description please see the ‘Data Records’ section provided in the supplemental R code^22^). All missing MAT and MAP values were estimated using a global air temperature and precipitation dataset provided by the Center for Climate Research at the University of Delaware^23^. The MAP and MAT were calculated based on the monthly precipitation and temperature between 1961 and 2015. Soil texture was grouped into coarse (sand, loamy sand, and sandy loam), medium (sandy clay loam, loam, silt loam, and silt), and fine (clay, sandy clay, clay loam, silty clay, and silty clay loam) textures based on the Cornell Framework^24^.

**Fig. 3:**
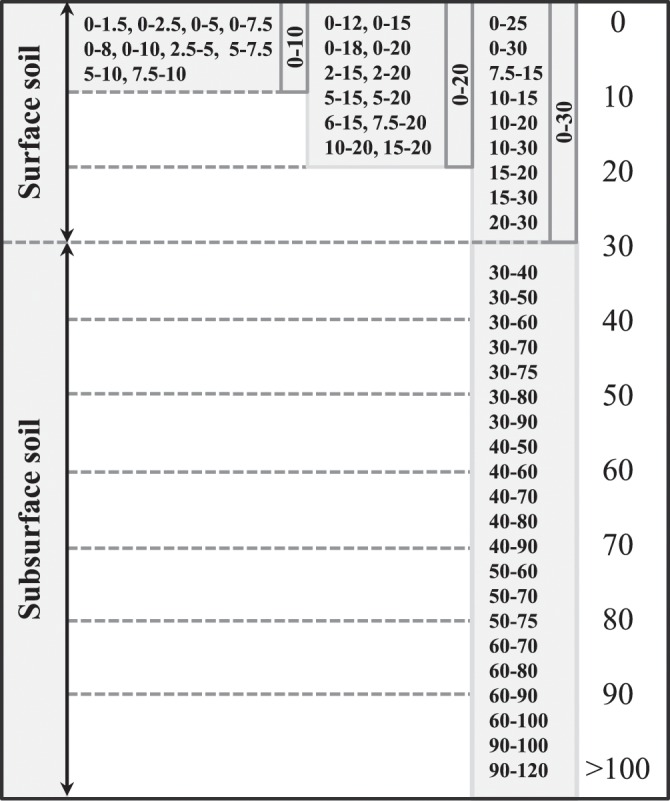
Diagram detailing how soil sampling depths were separated into 0–10 cm, 0–20 cm, 0–30 cm, and >30 cm groups.

The cash crops were grouped into corn, soybean, wheat, other monoculture, corn-soybean rotation (CS), corn-soybean-wheat rotation (CSW), and other rotation of more than two cash crops (ROT). The cover crops were grouped into broadleaf, grass, legume, mixture of two legumes (LL), mixture of legume and grass (LG), mixture of two cover crops other than LL or LG (MOT), and other mixtures of more than two cover crops (MTT). Soil sampling depths were grouped into 0–10 cm, 0–20 cm, 0–30 cm, and 30–100 cm (Fig. 3). It should be noted that the user can regroup the cash crop, cover crop, and soil sampling depth according their research objectives.

The number of replications and standard deviations (SD) were compiled from the publications when possible. When the studies reported standard error (SE), coefficient of variation (CV), or confidence interval (CI) rather than SD, SD was calculated using:

1 $$SD=SE\times \sqrt{n}$$

where *n* is the number of observations.

SD was calculated from CV as:

2 $$SD=CV\times mean$$

and from the CI as:

3 $$SD=\left|CI-mean\right|/(2{Z}_{a/2})\times \sqrt{n}$$

where Z_*α*/2_ is the *Z* score for a given level of significance, α. Z_*α*/2_ is equal to 1.96 when α = 0.05 and 1.645 when α = 0.10.

Soil organic carbon (SOC) data were reported as carbon stocks (Mg/ha). When applicable, SOC was calculated based on SOC concentrations (SOC_%_) and soil bulk density using:

4 $$SOC=SO{C}_{{\rm{ \% }}}\times h\times 100\times BD$$

where *h* represents soil sampling depth (meter), and *BD* represents soil bulk density (Mg/m^3^).

SOC sequestration rate (SOC_*seq*_) was calculated in terms of (Mg/ha/yr) using:

5 $${SOC}_{seq}=({SOC}_{cc}-{SOC}_{background}){\div}y$$

where SOC_*cc*_ is the soil carbon stocks under CC treatments (Mg/ha), SOC_*background*_ is the soil carbon stock either under background conditions or under the no cover crop controls (Mg/ha), and *y* represents years after CCs.

## Data Records

The data and R code can be downloaded in figshare^22^; there are two folders, named data and RScripts, when ‘SoilHealthDB.zip’ is unzipped. ‘SoilHealthDB_V1.xlsx’ in the data file currently includes 5,907 rows and 268 columns, which were retrieved from 321 papers (for the detailed reference list please refer to ‘References’ under ‘SoilHealthDB_V1.xlsx’^22^). Each column corresponds to one data point of either background information or soil health indicator, and each row includes as many as 42 comparisons between treatments and controls (if all soil health indicators have data). The names, attributes, and descriptions of the background information and soil health indicators are presented in Online-only Tables 1 and 2. It should be noted that different measurements and/or units may be involved in the same soil health indicator (e.g., soil total nitrogen, soil organic nitrogen, or soil inorganic nitrogen are reported in different papers to represent the soil nitrogen indicator, ID 5 in Online-only Table 2); therefore, it is important that measurement objectives, units, and other detailed descriptions are recorded in the comments columns. It should also be noted that for some soil health indicators (e.g., CH_4_ and N_2_O emission), we were only able to extract limited numbers of comparisons, which may restrain the ability of those data to be used in further analyses. ‘SoilHealthDB_V1.csv’ is a simplified version of ‘SoilHealthDB_V1.xlsx’, with only soil health background and indicator information kept (e.g., all the description sheets were not kept). There are two R scripts in the ‘RScripts’ folder: the ‘SoilHealthDB_quality_check.R’ script includes code for quality check of the ‘SoilHealthDB’, and the ‘functions.R’ script defines several functions, including one to generate the location of the site in ‘SoilHealthDB’. The SoilHealthDB_V1.csv file is to be used when running the R codes.

## Technical Validation

Quality control was performed to check the fidelity of the data to the original source. Each paper was carefully read at least twice, and special attention was paid to the tables, figures, and method sections, where most of the soil health indicator comparisons and background information were located. Before a new paper was extracted, we first used the bibliography database manager Mendeley to check whether it was a duplicate of previous papers (for details, please see the supplemental reference document). After the data extraction, we compared the digitized data against the tables or figures from the original paper once again to make sure the data were loaded correctly.

After the data extraction, we examined data quality using R (version 3.5.1)^25^. The formats of each column (numerical or string) were checked to correct any mistyping in the numerical columns (e.g., checking all soil health indicators and some background information columns like latitude and longitude). For each soil health indicator, we calculated the response ratio (RR), which is the value of treatment divided by the value of control, e.g., for cover crop studies RR = ln(*x*_*cc*_/*x*_*nc*_), where *x*_*cc*_ is the mean parameter value under cover crops and *x*_*nc*_ is the mean parameter value under no cover controls. We then plotted the frequency distribution of response ratio for each soil health indicator, and returned to the original articles to verify any extreme values that were identified in this process. We also visualized the data distribution for background columns that contained numeric values (e.g. latitude, elevation) and manually checked the outliers by validating them against the original papers. For the location of each site, we plotted the latitude and longitude by country and checked whether there were sites from a specific country that fell outside its border. For those sites, we checked the extracted latitude and longitude information with location information from the original paper (e.g., site name, country name). For some sites located near to coastal areas, a few sites were reported to exist in the sea, likely due to insufficient precision in reported values. For these sites, we slightly corrected the longitude and latitude to the nearest point on land.

### Linkages to external data sources

**Fig. 4:**
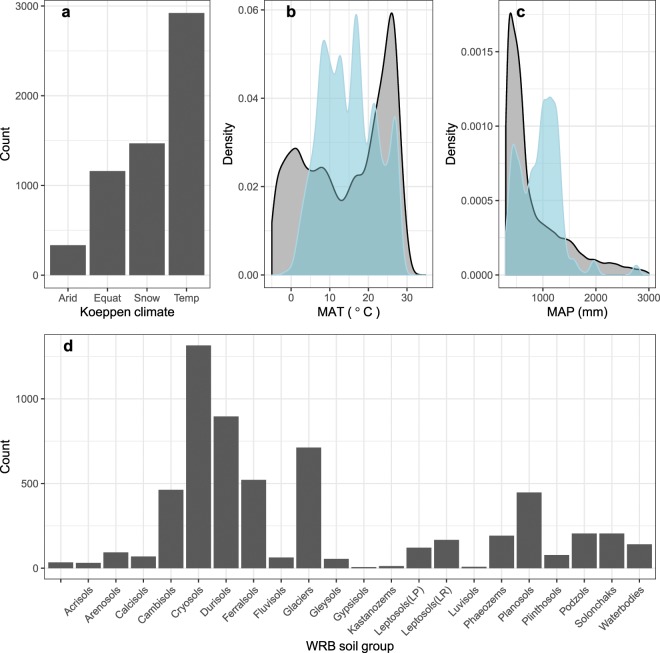
Representation of SoilHealthDB samples in different climate and soil types. Distributions of SoilHealthDB samples values across different parameters. Analyzed distributions include: (**a**) different climate types; (**b**) mean annual temperature (MAT); (**c**) mean annual precipitation (MAP); and (**d**) different WRB soil groups. Note that in (**a**) Equat – equatorial and Temp – temperate; in (**b**,**c**) the light blue represents samples from SoilHealthDB and gray represents global values from the Harmonized World Soil Database v1.2 (for details please see refs. ^26,27^).

The studies compiled thus far in *SoilHealthDB* rarely reported potentially important soil properties (e.g., cation exchange capacity, CEC) and background information (e.g., mean annual temperature, MAT, and mean annual precipitation, MAP). Similarly, some soil attributes such as soil taxonomy were classified differently between regions, making it difficult to compare this information. To resolve those issues, we associated our database with external data sources (by latitude and longitude; for details please see the code in the repository). We linked our data with Koppen^21^ classification (0.5° × 0.5° resolution), a global air temperature and precipitation dataset (0.5° × 0.5° resolution)^23^, and the Harmonized World Soil Database v1.2 (HWSD, 0.05° × 0.05° resolution)^26,27^. We then analysed all samples for their soil type, using the World Reference Base (WRB) classification system^26,27^, and for their climatic attributes (Fig. 4).

Samples from *SoilHealthDB* covered all four climate types, with the majority of sites located in temperate areas and relatively few sites located in arid areas (Fig. 4a). Sites within the *SoilHealthDB* had somewhat different distributions for MAT and MAP as compared to global distributions (Fig. 4b,c), in part because we only included locations with MAT between −5 °C and 35 °C so as to exclude climates not conducive to crop production. The MAT from *SoilHealthDB* sites followed an approximately normal distribution, with the most common temperatures occurring between 5 and 20 °C. In contrast the global MAT peaked between 20 and 30 °C. The majority of sites in *SoilHealthDB* had MAP between 500 and 1500 mm, while global MAP followed a gamma distribution with a greater proportion of area having <500 mm MAP. *SoilHealthDB* sites covered 21 out of 32 soil taxonomic groups in the WRB soil classification system^26,27^ (Fig. 4d).

**Fig. 5:**
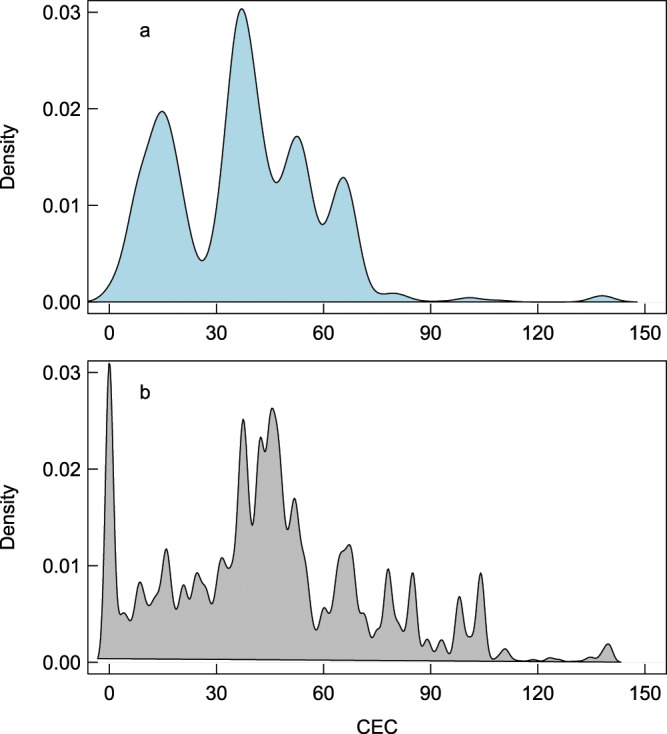
Distribution of cation exchange capacity (CEC) values. Densities are calculated for (**a**) samples from SoilHealthDB compared with (**b**) global soils, based on values obtained from the Harmonized World Soil Database v1.2.

Only 11 studies reported soil CEC (thus representing approximately 4% of all studies in *SoilHealthDB*), for a total of 54 independent records. There thus exists a paucity of direct CEC measurements in *SoilHealthDB*. However, we were able to estimate CEC for all sites using the HWSD soil database (Fig. 5a). Cation exchange capacity (CEC) distributions were similar between *SoilHealthDB* sites and the global HWSD soil database (Fig. 5b), suggesting that samples in the *SoilHealthDB* properly represent soil and climatic characteristics for regions conducive to agricultural production.

Finally, because attributes such as texture and CEC are important for interpreting soil health, we encourage future submissions to record these types of information to the extent possible. We also encourage use of the WRB taxonomy for all samples, as a way to enhance the global applicability of this database.

## Usage Notes

In the *SoilHealthDB*, the measurement objectives and units between each comparison (control vs. treatment within same row) will always be the same. However, each soil health indicator may have multiple measurement objectives and therefore involve multiple units (e.g., a researcher may measure soil total nitrogen in one site and measure organic nitrogen in another site). Detailed information about measurement objectives and units are recorded under the comments column. The user should always check the comments before data processing and analysis; otherwise, without data filtration and unit conversion only response ratios should be analysed. We recommend that users download and explore the database using the provided R code, as the code includes explanations and instructions. The user can contact the corresponding author with questions on understanding the code and using the data.

## Data Availability

All the data processing and data visualization were conducted using R (version 3.5.1)^25^. The source code is available on figshare^22^. The code is detailed with instructions for users. Generally, the *function.R* file (under *RScript* folder) defined several functions to obtain background information from external datasets, as well as the function to plot the samples spatial distribution (Fig. 1). The *SoilHealthDB_quality_check.R* file (under *RScript* folder) intends to check the data quality, and to explain how some soil health indicators are grouped based on the basic information. We also created a markdown file (*SHDB.Rmd*), which described the analysis and generated figures (Figs. 1, 4 and 5) for this study. All the code and data used are available in figshare^22^ and GitHub (https://github.com/jinshijian/SoilHealthDB).

## Online-only Tables

**Online-only Table 1:** Descriptions and attributes of background information in *SoilHealthDB*.

ID	Background indicator	Description
1	ExperimentID	Experiment ID number; please see details in Fig. 2
2.1	Author_F	First author’s Family name
2.2	Author_G	First author’s Given name
3	YearPublication	Paper publication year
4	SamplingYear	Sampling year, should be same or earlier than publication year
5	Journal	Name of journal in which paper was published
6	SiteInfor	Site name and detailed site information
7	Country	Country name where the study occurred
8	Latitude	Latitude of the site
9	Longitude	Longitude of the site
10	Elevation	Elevation of the site
11	Tannual	Annual average air temperature
12	MAT	Mean annual air temperature reported from paper (time span may differ from paper to paper) or from a global air temperature data^24^.
13	Pannual	Annual precipitation from paper
14	MAP	Mean annual precipitation (time span may differ from paper to paper) or from a global precipitation data^24^.
15	ClimateType	Study site’s climate type, following Koppen climate classification system^21^
16	YearsAfterCoverCrop	How many years cover crop applied before taking soil samples (e.g., experiment initiated at 1991, sample take at 1995, then fill with 5)
17	Duration	How many years the whole experiment lasted for (e.g., if an experiment started in 1990 and ended in 2000, the duration is 11 years)
18.1	CC_planting_date	Time that cover crops were planted
18.2	CC_termination_date	Time that cover crops were terminated
19	Time_Comments	Comments about cover crop planting period
20	SamplingDepth	Soil sampling depth, formatted as top-to-bottom, e.g., 10-to-20.
21.1	SamplingThickness	Difference between bottom and top sampling depths, e.g., for 10-to-20, the Sample Thickness is 10
21.2	SoilDepthGroup	Soil depth grouped based on sampling depth; see Fig. 3 for more details
21.3	SurfaceSubsurface	Surface or subsurface indicator; see Fig. 3 for more details
22	SoilBD	Bulk density
23.1	SandPerc	Percentage of sand
23.2	SiltPerc	Percentage of silt
23.3	ClayPerc	Percentage of clay
24.1	Texture	Soil texture
24.2	TextureGroup	Soil texture group (coarse, medium, and fine), based on the Cornell Framework of Soil Health Manual^25^.
25	SoilpH	Soil pH
26	BackgroudSOC	Background soil organic carbon; note that this column reports background soil carbon information (Unit is %), which is different than OC_C_concentration and OC_T that are reported in the response columns
27	SOC_NaturalVeg	Soil organic carbon of nearby natural vegetation land use (Unit is %)
28	SoilKsat	Soil saturated conductivity
29	SoilFamily	Soil family or soil group information; it should be noted that there are many soil classification system in the word, and studies from different countries may use different soil classification system
30.1	CoverCrop	Cover crop type; also referred to in literature as catch crop or green manure
30.2	CoverCropGroup	Cover crop grouped by function or family of cover crop; please see more details in the data file
31.1	GrainCrop	Grain crop type, also called cash crop
31.2	GrainCropGroup	Grain crop grouped by function or family of grain crop; please see more details in the data file
32	Landuse	Landuse type
33.1	Rotation_C	Type of rotation/crop sequence for control
33.2	Rotation_T	Type of rotation/crop sequence for treatment
33.3	Rotation_Diff	Whether the type of rotation/crop sequence differs between control and cover crop: yes or no
34.1	Tillage_C	Type of tillage for control
34.2	Tillage_T	Type of tillage for cover crop
34.3	Tillage_Diff	Whether type of tillage differs between control and cover crop: yes or no
34.4	TillageGroup_C	Tillage method grouped to CT, RT, or NT of control; for details please see the data file
34.5	TillageGroup_T	Tillage method grouped to CT, RT, or NT of treatment; for details please see the data file
35.1	Fertilization_C	Description about fertilization for control
35.2	Fertilization_T	Description about fertilization for treatment
35.3	Fert_Diff	Whether control and treatment applied different fertilizer levels: yes or no
36	ControlDescription	Control description (e.g., winter fallow, summer fallow, no cover crop, bare soil)
37.1	No_C	Number of plots in control
37.2	No_T	Number of plots in treatment
37.3	No_Supsample	Number of subsamples
38	ExperimentDesign	Experimental design: CRD, RCBD, split-design etc.
39	CCFreshBiomass	Fresh biomass of cover crop (returned to soil as green manure)
40	CCDryBiomass	Dry biomass of cover crop (returned to soil as green manure)
41	CNOfCoverCrop	Carbon to nitrogen ratio of the cover crop dry biomass (determine quality of green manure)
42	CCTermination	Method of killing cover crop
43	Conservation_Type	Type of conservation management: cover crop (CC), no-tillage (NT), organic farm (OF), agro-forestry system (AF)
44	Conservation_ Description	More details or descriptions on conservation agriculture method
45.1	CEC	Soil cation exchangeable capability
45.2	CEC_unit	Unit of soil cation exchangeable capability
46	Other	Other meta/background information about the publication

**Online-only Table 2:** Description and attributes of soil health indicators in the *SoilHealthDB*.

ID	Indicator	Description	Comments
1	Biomass	Biomass of cash crop excluding yield (e.g., stem, leave, root)	Units vary, but are in kg/ha if not otherwise indicated
2	Yield	Yield of grain (cash) crop	Units vary, but are in kg/ha if not otherwise indicated
3	BD	Soil bulk density	Units = g/cm^3^
4.1	SOC_Conc	Soil organic carbon concentration (in unit of %)	SOC = SOM/1.72
4.2	SOC_Stock	Soil organic carbon stock	Units = Mg/ha; when not reported, it can be calculated by Eq. (4)
4.3	SOC_SEQ	Soil organic carbon sequestration rate	Units = Mg/ha/cm/yr; can be calculated by either Eq. (5) or (6)
5	N	Soil Nitrogen	Units vary
6	P	Soil Phosphorus	Units vary
7	K	Soil Potassium	Units vary
8	pH	Soil pH	No units
9	CEC	Soil cation exchange capability	Units vary
10	EC	Soil electric conductivity	Units vary
11	BS	Soil base Saturation	Units vary
12	Aggregation	Soil aggregation	There are multiple ways to measure and report soil aggregation; units vary
13	Porosity	Soil porosity	Units vary
14	Penetration	Soil penetration resistance	Units vary
15	Infiltration	Soil infiltration rate	Units vary
16	Ksat	Field saturated hydraulic conductivity	Units vary
17	Erosion	Soil erosion or wind erosion	Units vary
18	Runoff	Runoff	Units vary
19	Leaching	Soil nutrient leaching	Units vary
20	ST	Soil temperature	°C
21	SWC	Soil water content	Units vary
22	AWHC	Available water hold capacity	Units vary
23	Weed	Weeds in the cropland	Units vary
24	Diseases	Diseases of the cropland	Units vary
25	Pests	Pests in the cropland	Units vary
26	SoilFauna	Earthworms, athropods, nematodes	Units vary
27	Fungal	Bacteria, fungi, mycorrhizi in the soil	Units vary
28	O-Microbial	Other microbial indicators	Units vary
29	Enzyme	Enzyme activity	Beta-glucosidase activity and phenol oxidase; units vary
30	Cmin	Soil mineralizable carbon	Units vary
31	Nmin	Soil mineralizable nitrogen	Units vary
32	N_2_O	Soil N_2_O efflux	Units vary
33	SIR	Soil substrate-induced respiration	Units vary
34	CO_2_BTest	Soil CO_2_ burst test respiration	Units vary
35	CO_2_	Soil respiration	Units vary; some literature calls this CO_2_ efflux or CO_2_ flux
36	CH_4_	Soil CH_4_ emission	Units vary
37	MBC	Microbe biomass carbon	Units vary
38	MBN	Microbe biomass nitrogen	Units vary
39	Microelement	Mn, Zn, Cu etc.	We did not record the actual data; instead, we labelled with 9999 if a paper reported microelements
40	SQI	Soil quality indicator, soil health indicator	We did not record the actual data; instead, we labelled with 9999 if a paper reported SQI
41	ESS	Ecosystem services indicator	We did not record the actual data; instead, we labelled with 9999 if a paper reported ESS
42	Texture	Cover crop effect on soil texture compared with control	We do not record the actual data; instead, we labelled with 9999 if a paper reported Texture
	Other_ comments	Other comments about soil health indicators (e.g., notes about indicators which currently not included in above 42 indicators)	

Note that in the data sheet, each indicator has 5 columns, recording information for mean of control, mean of treatment, standard deviation (SD) for control, SD for treatment, and comments.
